# A Self Inserted Unusual Foreign Body “An Entire Pencil” in a Male Urethra and Bladder: A Case Report

**DOI:** 10.7759/cureus.25198

**Published:** 2022-05-22

**Authors:** Mohammed M Ahmed, Nishath A Ahmed, Vivek Sharma

**Affiliations:** 1 General Surgery, Shyam Shah Medical College, Rewa, IND; 2 Pediatrics, Dr. B.R. Ambedkar Medical College & Hospital, Bangalore, IND; 3 Urology, Super Speciality Hospital Rewa, Rewa, IND

**Keywords:** rare foreign body, emergency, intact pencil, urinary bladder, urethra

## Abstract

The incidence of lower urinary tract foreign body insertions is low. The motives for the insertion of objects are complex to comprehend and could be a result of exotic impulses, psychometric problems, or sexual curiosity. Here we discuss a case of a 21-year-old male who came to the emergency room with complaints of a painful protrusion from the perineum and a history of insertion of an unusual foreign body in the form of an approximately 15cm long pencil that was inserted out of sexual curiosity to achieve autoerotism which was impacted in the posterior urethra and the bladder. Diagnosis in such cases can be achieved by proper history taking, conducting a thorough physical examination, and with use of appropriate imaging. The treatment options vary between minimally invasive procedures such as endoscopic removal and surgical treatment, with the former being used more often and the latter being done when the minimally invasive procedures are not able to remove the foreign body or when urethral or bladder injuries are expected in doing so. The complete case is discussed in detail to derive the proper management strategy in such rare cases.

## Introduction

Lower urinary tract foreign bodies are not often encountered, but many such cases have been published. These are commonly inserted secondary to curiosity, iatrogenic, or for autoerotic stimulation [1]. The materials inserted vary from wires, batteries, screws, and pens to weird ones, including animals, with the snake being an example [2]. The presentation varies, and so does the kind of foreign body inserted. The commonest cause of urethral foreign body self-insertion is autoerotism. The presentation is typically delayed because of the embarrassment that the patient experiences [3]. In most cases, nonetheless, the inserted foreign body results in extreme pain, urinary tract infections, and hematuria [4]. Techniques employed in the extraction of these foreign bodies have to be simple and should cause no undue trauma to the urethra and the bladder [1].

Despite the presence of comparable literature [1,5], the case that we discuss here of an entire pencil in toto as urethral and bladder foreign body is a rarity.

## Case presentation

**Figure 1:**
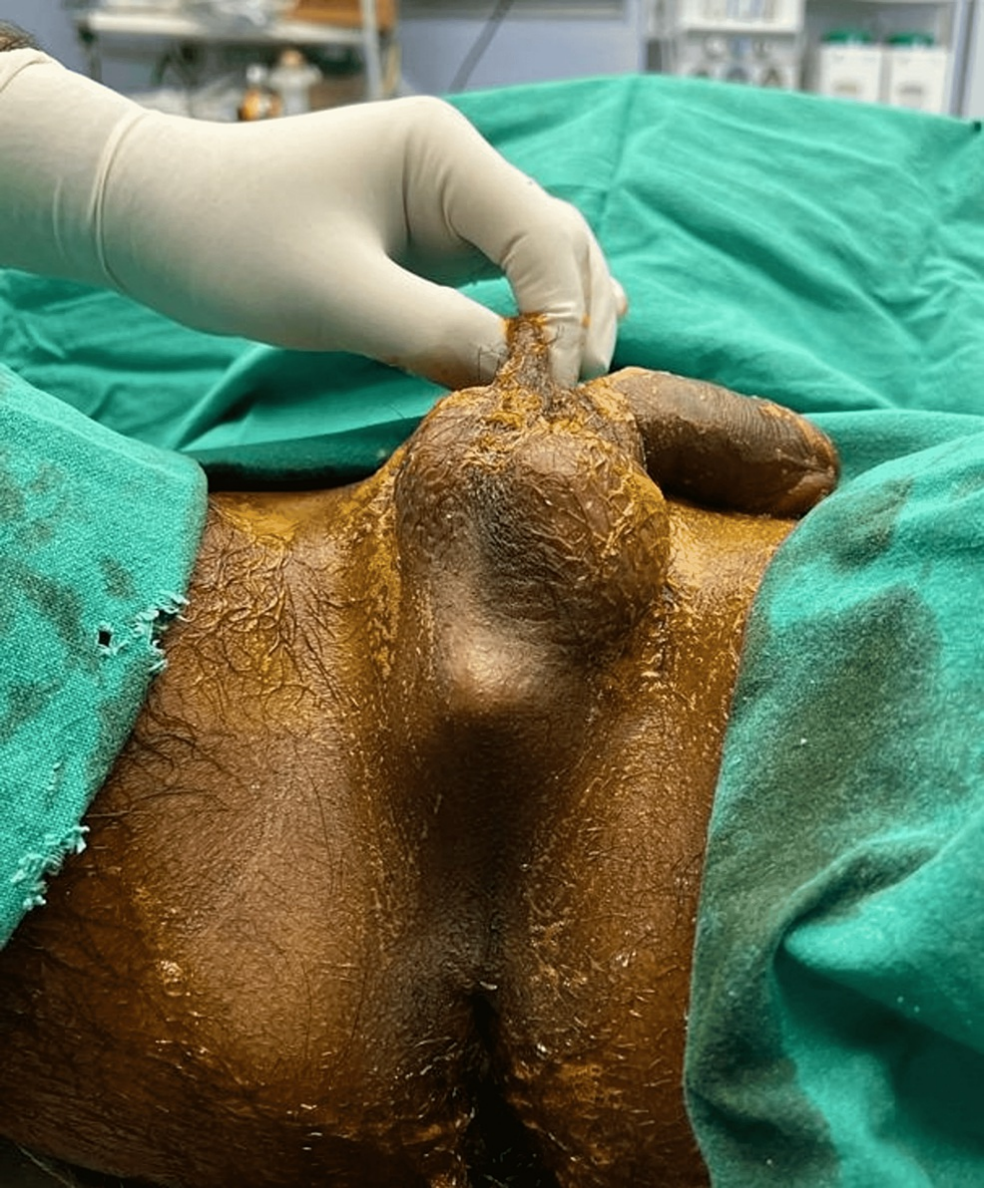
Local examination under spinal anesthesia revealed the protrusion caused by the foreign body

A 21-year-old male presented to the emergency department with complaints of a painful protrusion from his perineum and dysuria. No other urinary symptoms were noted. Detailed history revealed that he had self-inserted an approximately 15 cm long pencil with the blunt end inwards about six hours prior for autoerotic stimulation. There was no formal history of any previous psychiatric disorder. The patient revealed that he had previously also performed the same act about 3-4 times over the past three months using the same pencil. Each time the patient would insert the pencil, the blunt end was inwards, and he would insert it until two-thirds of the pencil was inside. On clinical examination, the urethral orifice was normal, with no part of the foreign body seen or felt here. The bulge caused by one end of the foreign body was clearly visible and palpable in the perineum in the posterior urethra (Figure 1). On per rectal examination, it was palpable in the bladder as well.

**Figure 2:**
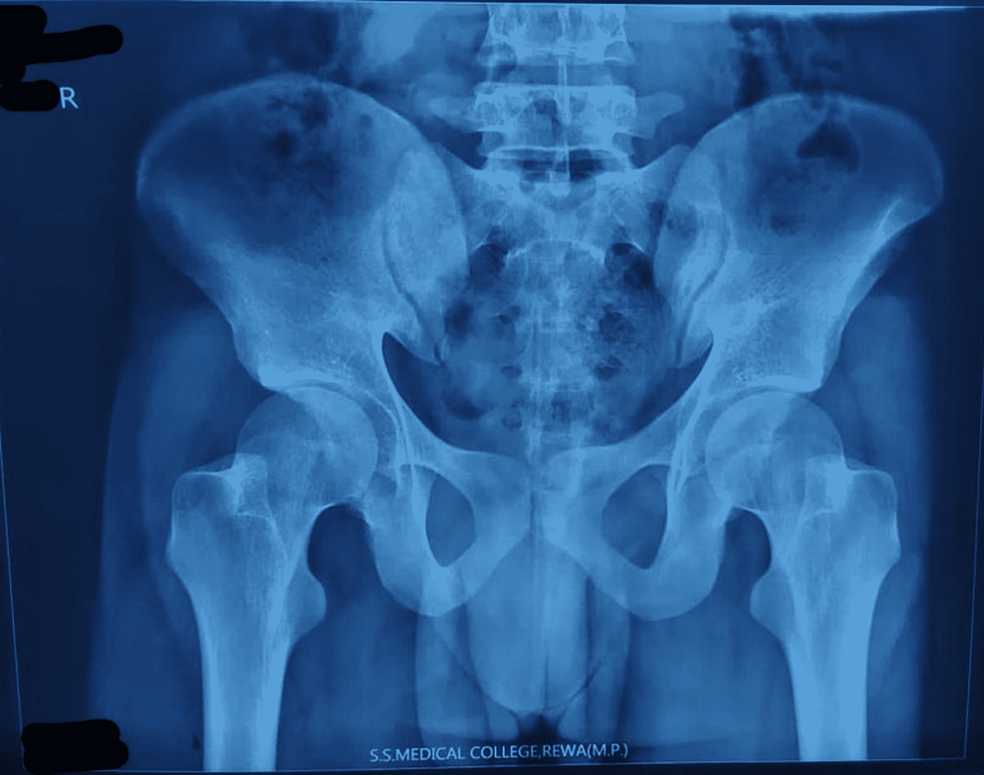
Preoperative pelvic X-ray

A pelvic radiograph did not reveal a clear-cut and well-demarcated foreign body (Figure 2).

**Figure 3:**
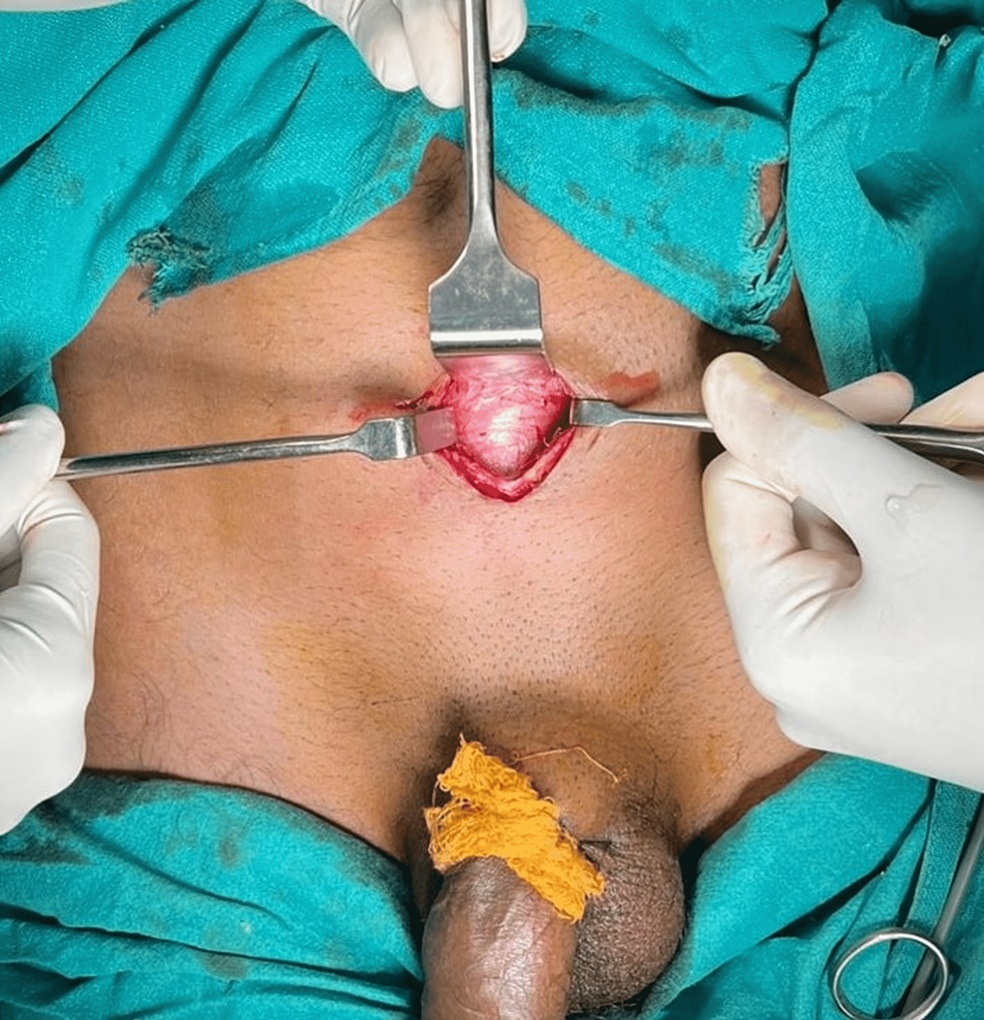
Suprapubic incision given and dissection done to reach the urinary bladder as seen

**Figure 4:**
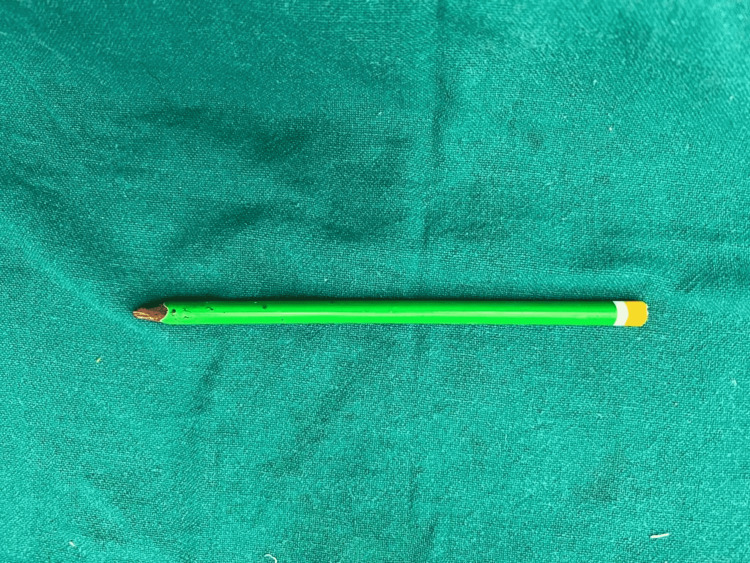
The foreign body (pencil) removed

The decision was made to proceed with a diagnostic cystoscopy under spinal anesthesia, which revealed no stricture, narrowing, or mucosal injuries to the anterior urethra. The foreign body was found lodged in the membranous urethra with no obvious penetrating type of injury to the urethra and extending proximally via the prostatic urethra through the external sphincter into the bladder (Video 1). Both the ureteric orifices were normal, and the bladder wall was intact. Endoscopic extraction via the urethra seemed impossible because the pencil was snuggly lodged with one end buried in the membranous urethra and the other in the bladder wall. Any attempt to mobilize the foreign body endoscopically would only result in further trauma to the bladder wall, prostate, or the urethra. So to prevent this and the resulting long-term complications that would ensue, the decision to go for an open suprapubic, transvesical extraction of the entire pencil was taken. The procedure was carried out successfully (Figure 3 shows the incision given, Figure 4 shows the pencil retrieved, and Video 2 shows the extraction).

**Video 1:** Diagnostic cystoscopy showing the foreign body lodged in the posterior urethra and bladder

**Video 2:** Suprapubic transvesical open retrieval of the foreign body

 

**Figure 5:**
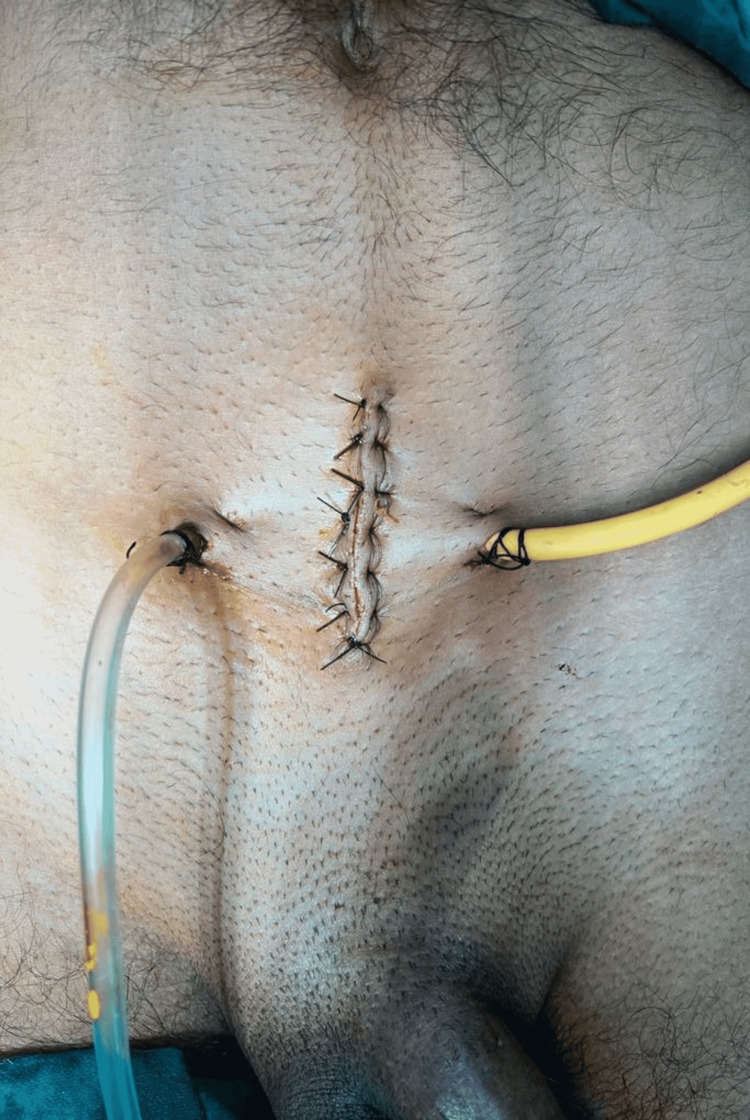
Postoperative photo showing the drain and the suprapubic catheter placed

The bladder was repaired in a standard fashion. Per urethral silicon catheter and a suprapubic catheter along with a drain in the space of Retzius were placed (Figure 5). The drain was removed on POD 3 before discharge, and in subsequent follow-ups the per urethral and suprapubic catheters were removed as well. A psychiatric evaluation was advised on discharge.

## Discussion

Causes of foreign bodies in the lower urinary tract include psychological, iatrogenic during urological procedures, traumatic aspects and migration from other organs [6]. Various psychological factors such as exotic impulse, mental illness, borderline personality disorder, sexual curiosity, sexual practice while intoxicated, and so on result in the self-insertion of foreign bodies in the lower urinary tract. Among these, the most common motive for foreign body insertion in the lower urinary tract is sexual or erotic in nature, such as masturbation or other forms of sexual variation or gratification [2,7]. Various options are reported for the treatment of bladder foreign bodies, for example, endoscopic, laparoscopic, percutaneous, radiological, and open surgery. In some cases, a combination of techniques is required. Ultimately, the availability of surgical instrumentations and the urologist's experience play an important role [5]. Urethral foreign bodies which are inserted via the urethral orifice usually migrate into the bladder by being pushed further into the urethra in the process of removing them or by involuntary perineal muscle contraction. Migration through the bulbous urethral curvature without significant injuries is surprising and still not sufficiently explained [6]. Urethral self-insertion of foreign bodies may be complicated when the inserted object migrates to the proximal urethra or bladder and cannot be retrieved [7]. 

The success rate of endoscopic management is high, and it has the added advantage of reducing the urethral and bladder injuries. However, open procedures become inevitable in certain cases with perineal urethrotomy or suprapubic cystostomy being the options available according to the nature of the foreign body [6].

In our case, the foreign body (a pencil) was impacted in the posterior urethra and the bladder and could not be retrieved by cystoscopic maneuvers. Nonetheless, the cystoscopic examination noted no injury or a breach in the bladder, no gross urethra injury, bilateral ureteric orifices were intact, prostate and bladder neck were normal. Open cystolithotomy was decided upon to access and retrieve the pencil. By taking this decision, injury to the bladder or urethra was avoided.

With prompt diagnosis and accurate management, lower urinary tract foreign bodies can be removed successfully. But, complications such as stone or fistula formation and infections do occur if foreign bodies remain entrapped for extended durations of time. Certain case reports have also documented complications as severe as sepsis and uremia, resulting in the deaths of the patients [8].

Finally, the patient was advised for psychiatric evaluation, but he refused the consultation.

## Conclusions

This particular case highlighted the presentation, possible management protocols and the definitive treatment that was given in such a rare clinical scenario. The treatment approach is crucial in such cases, which includes the prevention of further injury to the urethra and bladder, infection prevention and monitoring the delayed complications. Finally, the complete psychological evaluation of the patient to prevent further such episodes is equally important. 
